# Investigation of TGF*β*1-Induced Long Noncoding RNAs in Endothelial Cells

**DOI:** 10.1155/2016/2459687

**Published:** 2016-04-10

**Authors:** Krishna K. Singh, Pratiek N. Matkar, Adrian Quan, Laura-Eve Mantella, Hwee Teoh, Mohammed Al-Omran, Subodh Verma

**Affiliations:** ^1^Division of Vascular Surgery, Keenan Research Centre for Biomedical Science, St. Michael's Hospital, Toronto, ON, Canada M5B 1W8; ^2^Division of Cardiac Surgery, Keenan Research Centre for Biomedical Science, St. Michael's Hospital, Toronto, ON, Canada M5B 1W8; ^3^Department of Surgery, University of Toronto, Toronto, ON, Canada M5T 1P5; ^4^Institute of Medical Science, University of Toronto, Toronto, ON, Canada M5S 1A8; ^5^Division of Cardiology, Keenan Research Centre for Biomedical Science, St. Michael's Hospital, Toronto, ON, Canada M5B 1W8; ^6^Department of Pharmacology and Toxicology, University of Toronto, Toronto, ON, Canada M5S 1A8; ^7^Division of Endocrinology & Metabolism, Li Ka Shing Knowledge Institute, St. Michael's Hospital, Toronto, ON, Canada M5B 1W8; ^8^Department of Surgery, King Saud University and the King Saud University-Li Ka Shing Collaborative Research Program, Riyadh 12372, Saudi Arabia

## Abstract

*Objective*. To evaluate the relationship between TGF*β* signaling and endothelial lncRNA expression.* Methods.* Human umbilical vein endothelial cell (HUVECs) lncRNAs and mRNAs were profiled with the Arraystar Human lncRNA Expression Microarray V3.0 after 24 hours of exposure to TGF*β*1 (10 ng/mL).* Results*. Of the 30,584 lncRNAs screened, 2,051 were significantly upregulated and 2,393 were appreciably downregulated (*P* < 0.05) in response to TGF*β*. In the same HUVEC samples, 2,148 of the 26,106 mRNAs screened were upregulated and 1,290 were downregulated. Of these 2,051 differentially expressed upregulated lncRNAs, MALAT1, which is known to be induced by TGF*β* in endothelial cells, was the most (~220-fold) upregulated lncRNA. Bioinformatics analyses indicated that the differentially expressed upregulated mRNAs are primarily enriched in hippo signaling, Wnt signaling, focal adhesion, neuroactive ligand-receptor interaction, and pathways in cancer. The most downregulated are notably involved in olfactory transduction, PI3-Akt signaling, Ras signaling, neuroactive ligand-receptor interaction, and apoptosis.* Conclusions.* This is the first lncRNA and mRNA transcriptome profile of TGF*β*-mediated changes in human endothelial cells. These observations may reveal potential new targets of TGF*β* in endothelial cells and novel therapeutic avenues for cardiovascular disease-associated endothelial dysfunction.

## 1. Introduction

Transforming growth factor-*β* (TGF*β*) belongs to a large superfamily of linked proteins, comprising activins, bone morphogenetic proteins (BMPs), growth/differentiation factors, and anti-Müllerian hormone [1] that regulates proliferation, differentiation, migration, and survival in diverse cell populations depending on the cell type [2]. TGF*β*1, TGF*β*2, and TGF*β*3 are the most common of the isoforms that are involved in these functions [3]. Prior to binding to its specific type I and type II serine/threonine kinase receptors, the latent form of TGF*β* is activated by proteases or thrombospondin. It is well documented that TGF*β* signaling involves one TGF*β* type II receptor and two distinct TGF*β* type I receptors, that is, the endothelium limited activin receptor-like kinase (ALK1) and the largely expressed ALK5. Activated ALK5 after ligand binding transduces signals from the membrane to the nucleus via phosphorylation of a specific subset of intracellular effectors termed Smads [3, 4]. While ALK1 activation phosphorylates Smad1, Smad5, and Smad8, ALK5 mediates Smad2 and Smad3 phosphorylation. The heteromeric complex of phosphorylated Smad2/Smad3 with Smad4 then translocates to the nucleus, where, together with various transcriptional regulators, it leads to the transcription of a wide array of target genes [5, 6].

Several* in vivo* studies have shown that interfering with the components of the TGF*β*1 signaling pathway, including TGF*β*1 [7], TGF*β*R-II [8], ALK5 [9], endoglin [10], ALK1 [11], or Smad5 [12], through gene targeting results in extreme vascular anomalies in mice as illustrated by enlarged vessels and defective differentiation of smooth muscle cells. Depending on the experimental conditions and animal models, TGF*β*1 has been also shown to function as an inhibitor or a promoter of angiogenesis [13, 14]. Given its multifunctional role in cellular processes, disturbed TGF*β*1 signaling is notably evident in various human disorders [15, 16]. Evidence for how TGF*β*1 contributes to the advancement of tumors is conflicting and appears to be dependent on the developmental stage of the tumor. TGF*β*1 acts as an inhibitor of proliferation during the initial stages of tumor development. However, upon attenuation of this antiproliferative signal, tumor cells often secrete great amounts of TGF*β*1 which promote cell invasion, epithelial-to-mesenchymal transition (EMT) metastasis, and angiogenesis which collectively establish a growth-supportive tumor microenvironment [4, 17, 18].

In recent years, the long noncoding RNAs (lncRNAs) have emerged as regulators and potential therapeutic targets for a wide variety of physiological and pathological processes [19, 20]. Typically, lncRNAs are transcripts greater than 200 nucleotides that lack an open reading frame and protein-coding ability. Although the lncRNAs are not as well conserved as protein-coding genes and microRNA, increasing evidence suggests that lncRNAs are involved in a variety of cellular functions like proliferation, survival, migration, invasion, angiogenesis, and differentiation and could serve as alternative therapeutic targets [21–26]. MALAT1 (metastasis associated lung adenocarcinoma transcript 1), which is amongst the most abundant and highly conserved lncRNAs, exhibits specific nuclear localization, developmental regulation, and dysregulation in cancer, all of which are indicative of its critical role in multiple biological processes [27]. MALAT1 is an important mediator of TGF*β* signaling and may represent a promising therapeutic option for suppressing bladder cancer progression [28]. MALAT1 is highly expressed in endothelial cells and loss of MALAT1 tips the balance from a proliferative to a migratory endothelial cell phenotype* in vitro* and reduces vascular growth* in vivo* [29].

To date, the nuances underlying the transcriptional regulation of lncRNAs by TGF*β*1 in endothelial cells remain unexplored. The goal of the current study was to profile the changes in lncRNA expression in association with TGF*β*1 signaling in endothelial cells that may provide insights into regulation of endothelial function by TGF*β*1-associated lncRNAs. This approach also allowed us to identify novel lncRNA targets and associated pathways of TGF*β*1 in endothelial cells.

## 2. Materials and Methods

### 2.1. Cell Culture

Human umbilical vein endothelial cells (HUVECs, Lonza) were cultured in endothelial cell growth medium-2 (EGM*™*-2 Bulletkit*™*; Lonza) supplemented with growth factors, serum, and antibiotics at 37°C in humidified 5% CO_2_. Confluent HUVECs were split into 6 and maintained in 6-well plates for 24 hours in the absence (3 plates) or presence (3 plates) of recombinant TGF*β*1 (10 ng/mL; R&D Systems).

### 2.2. Microarray Profiling

Total RNA was isolated using TRIzol*™* (Invitrogen) reagent and quantified with the NanoDrop ND-1000 spectrophotometer. RNA integrity was confirmed by standard denaturing agarose gel electrophoresis. The expression profile of 30,584 human lncRNAs and 26,106 protein-coding transcripts was conducted with the Arraystar Human LncRNA Microarray V3.0. Sample labeling and array hybridization were performed on the Agilent Array platform. Briefly, total RNA from each sample was amplified and transcribed into fluorescent cRNA (Arraystar Flash RNA Labeling Kit, Arraystar) before 1 *μ*g of each labeled cRNA was hybridized onto the microarray slide. The hybridized arrays were washed, fixed, and scanned with the Agilent DNA Microarray Scanner (Product^#^ G2505C). The acquired array images were analyzed with the Agilent Feature Extraction software (version 11.0.1.1). Quantile normalization and subsequent data processing were performed with the GeneSpring GX v11.5.1 software package (Agilent Technologies). *P* values for the differentially expressed genes were determined with the *t*-test and adjusted for multiple testing with the Benjamini Hochberg method to minimize the false discovery rate. Volcano plot filtering, set at a threshold of ≥2.0-fold, was used to screen for lncRNAs and mRNAs that exhibited significantly different (*P* < 0.05; unpaired *t*-test) expression levels in the two study groups. Pathway analysis was based on the current Kyoto Encyclopedia of Genes and Genomes (KEGG) database. Gene ontology (GO) analysis was performed with the topGO package of bioconductor system.

## 3. Results

### 3.1. Quality Assessment of LncRNAs and mRNAs Data

The 6 samples evaluated had a 2 : 1 intensity ratio for their 28S : 18S rRNA bands and OD260/OD280 ratios of >1.8 thereby verifying RNA integrity, purity, and concentration (Supplementary Figure  1 in Supplementary Material available online at http://dx.doi.org/10.1155/2016/2459687). Box-and-Whisker plots constructed to visualize the distribution of the fluorescent intensities revealed very similar normalized log 2 ratios for both lncRNA and mRNA and accordingly comparable quality of the array data, across the board (Supplementary Figure  1).

**Figure 1:**
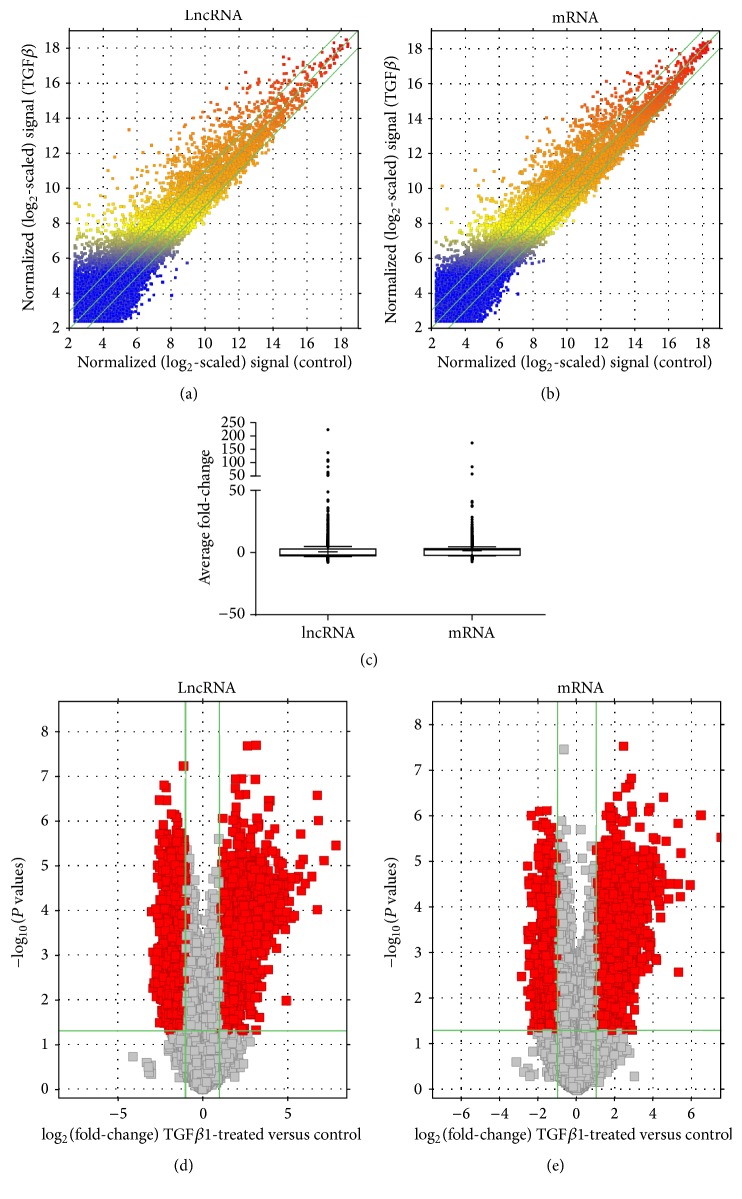
LncRNA and mRNA expression profiles in HUVECs exposed to TGF*β*1 (10 ng/mL) versus control. (a and b) Scatter plots comparing the variation in lncRNA and mRNA expression. The values plotted are the averaged normalized signal values (log 2 scaled) for the control (*x*-axis) and the TGF*β*1 treatment (*y*-axis) groups. The green lines indicate fold-change. LncRNAs and mRNAs above the top green line and below the bottom green line exhibit at least a 2.0-fold difference between the two study groups. (c) Box-and-Whisker plots (10th and 90th percentiles) showing average fold-change of lncRNAs and mRNAs. Median intensity is denoted with a “−” sign and mean intensity is denoted with a “+” sign. (d and e) Volcano plots detailing magnitude of expression difference. The vertical green lines correspond to 2.0-fold upregulation and 2.0-fold downregulation of expression. The horizontal green line indicates a *P* value of ≤0.05. Red points represent lncRNAs and mRNAs with statistically significant differential expression (fold-change ≥ 2.0, *P* ≤ 0.05).

Scatter plots provided a profile of HUVEC lncRNAs (Figure 1(a)) and mRNAs (Figure 1(b)) that were upregulated, downregulated, or unaffected by exposure to TGF*β*1 treatment. Overall, the average fold-changes of lncRNAs and mRNAs differentially expressed under the study conditions were similar (Figure 1(c)). Subsequent volcano plot filtering uncovered 2,051 significantly upregulated and 2,393 significantly downregulated lncRNAs in HUVECs cultured with TGF*β*1 relative to control samples (Figure 1(d); *P* < 0.05; Supplement Tables  A and B). LncRNAs that demonstrated the greatest differences in expression ranged from 177 bp to 17.85 kb. Specifically, MALAT1 (RNA length: 8,708 bp, chromosome 15) was the most upregulated lncRNA and AC144521.1 (RNA length: 919 bp, chromosome 3) was the most downregulated in HUVECs subjected to TGF*β*1 treatment.  Table 1 lists the 10 most up-/downregulated lncRNAs depending on the fold-change expression. TGF*β*1 treatment associated changes at the transcript level were also noted amongst 3,436 mRNAs with 2,148 upregulated and 1,290 downregulated (Figure 1(e); *P* < 0.05; Supplement Tables  C and D).

### 3.2. LncRNA Chromosomal Distribution and Subtype Analysis

**Figure 2:**
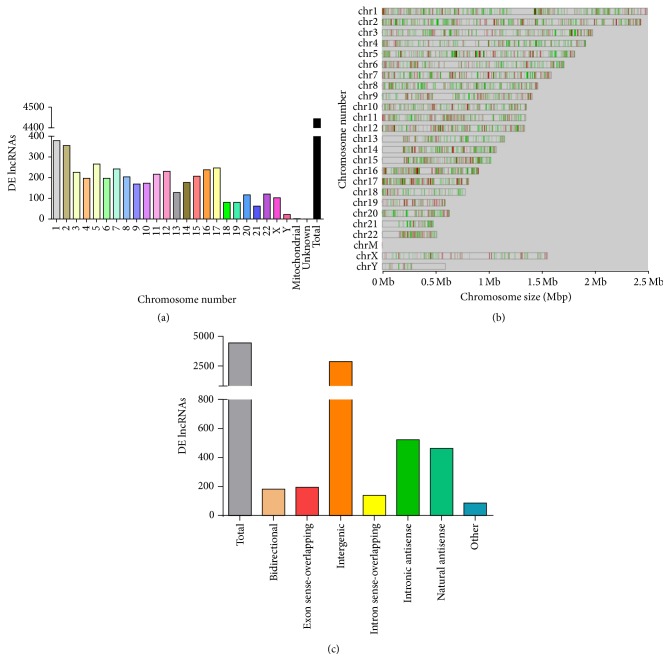
Distribution, location, and classification of differentially expressed lncRNAs in HUVECs exposed to TGF*β*1 (10 ng/mL) versus control. Demonstration of (a) numbers and (b) chromosomal location of differentially expressed (DE) lncRNAs on different chromosomes. (c) Bar graph representing types of differently expressed lncRNAs, depending on their genomic location.

Supplementary Figure  2 shows the dendrograms generated for hierarchical analysis of clustered lncRNAs and mRNAs that were differentially expressed in HUVECs cultured in media with TGF*β*1 in comparison to controls. Although lncRNAs modulated by TGF*β*1 treatment were abundant and found on every human chromosome, most were located on chromosomes 1, 2, and 17 (Figure 2(a)). Further probing revealed that while these differentially expressed lncRNAs are expressed along the entire length of the chromosomes, there is a notable clustering of lncRNAs (Figure 2(b)). LncRNA subgroup analysis, which helps identify the functional relationship between lncRNAs and their associated protein-coding genes, demonstrated that the majority (~50%) of lncRNAs were intergenic in origin followed by intron and natural antisense lncRNAs (Figure 2(c)). We also identified bidirectional, exon sense-overlapping, and intron sense-overlapping lncRNAs (Figure 2(c)).

### 3.3. LncRNAs and Associated Protein-Coding Transcripts

**Figure 3:**
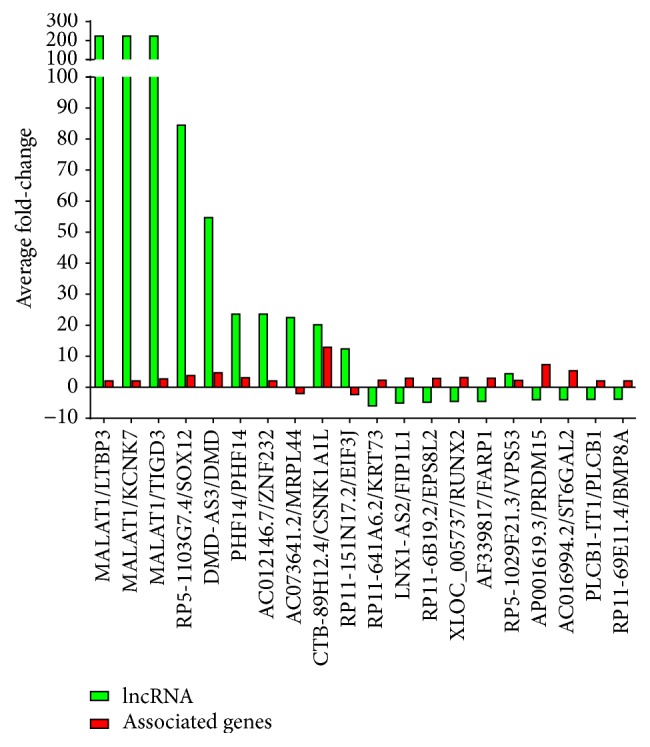
Network coexpression and bioinformatics analyses of samples from HUVECs exposed to TGF*β*1 (10 ng/mL) versus control. Representation of differentially expressed lncRNAs and associated genes with respect to fold-change. Eight significantly upregulated and 10 downregulated lncRNAs with known target genes were selected for presentation in the figure.

We conducted additional profiling to gather insight into differentially expressed lncRNAs and associated protein-coding transcripts. The fold-change calculated for the top 10 highly up-/downregulated lncRNA with known associated protein-coding genes is summarized in Figure 3. Interestingly, MALAT1, which is highly expressed in endothelial cells [29] and is an important mediator of TGF*β* signaling [28], was the most upregulated lncRNA after TGF*β*-stimulation (Figure 3). The protein-coding genes LTBP3, KCNK7, and TGD3, which are adjacent to MALAT1 on chromosome 15 [27], were also significantly upregulated (Figure 3). Of note, 9 of the 20 lncRNAs demonstrated a direct correlation in fold-change with its associated mRNA, whereas the remaining 11 displayed an inverse correlation. Inverse relation was mainly observed for the downregulated (9 out of 10) lncRNAs (Figure 3).

### 3.4. Bioinformatics Analyses

Pathway analysis with the current KEGG database yielded several pertinent findings (Tables 2 and 3). In brief, mRNAs upregulated in response to TGF*β*1 treatment are involved in hippo signaling, Wnt signaling, focal adhesion, neuroactive ligand-receptor interaction, and cancer-associated pathways (Table 2). The most downregulated mRNAs are notably involved in olfactory transduction, PI3K-Akt signaling, Ras signaling, neuroactive ligand-receptor interaction, and apoptosis (Table 3).

Bioinformatics GO analyses grouped the differentially expressed mRNAs under the following three categories: biological processes, cellular component, and molecular function. GO terms most broadly associated with upregulated mRNAs were biological function, protein binding, and signalling (Table 4). GO terms associated with downregulated mRNA were mainly enriched in cell, response to stimulus, and multicellular organism process (Table 4).

## 4. Discussion

The underlying dogma of molecular biology for the last few decades has been that the purpose of RNA is to direct the assembly of proteins from amino acids through translation. A few exceptions to this paradigm are ribosomal RNA and transfer RNA which are functional RNA macromolecules that do not encode protein. A large proportion (>80%) of the human genome is transcribed, but protein-coding transcripts account for only ~2% of whole transcriptome [30]. This suggests that the majority of the genomes are transcribed as non-protein-coding RNAs. Among noncoding RNAs, a novel class of noncoding RNAs, which stretch more than 200 nucleotides and are termed long noncoding RNAs (lncRNAs), has recently emerged [31]. Evidence to date suggests that the mechanisms underlying gene regulation by lncRNAs are highly complex and involve both inhibition and activation of gene expression [32].

The growing appreciation of the multitude of mechanisms, functions, and types of lncRNAs has set off a research tsunami to clarify the involvement of lncRNAs in the etiology of disease states. Although there have been reports demonstrating that lncRNAs are dysregulated in several human diseases, it has yet to be confirmed that these molecules can act independently to drive the progression of said pathologies [33]. At present, the strongest association lies with cancer [34] where altered expression of several lncRNAs has been documented [35, 36]. LncRNA PCAT-1 which is a target of histone-modifying PRC2 complex bearing both oncogenic and tumor-suppressive features was found to promote cell proliferation [37]; antisense noncoding RNA in the INK4 locus (ANRIL; also known as CDKN2BAS) is upregulated in prostate cancer and implicated in tumor suppression [38]; HOTAIR upregulation is associated with poor prognosis in pancreatic [39], colorectal [40], liver [41], gastrointestinal [42], and breast [43] cancers and likely also contributes to increased metastasis [43] of these cancer types. MALAT1 was one of the first lncRNAs to be implicated in cancer and a series of studies have established its potential importance as a biomarker and potential therapeutic target for cancer metastasis [44]. Increased expression of MALAT1 is observed in lung, breast, colon, cervical, colorectal, ovarian, gastric, and other cancer types [44]. Mechanistically, MALAT1 affects the transcriptional and posttranscriptional regulation of cytoskeletal and extracellular matrix genes [45]. A similar function has been postulated for lincRNA-p21 (named for its vicinity to the CDKN1A/p21 locus) in cancer, which functions as a repressor in p53-dependent transcriptional responses particularly on genes regulating apoptosis, possibly by directing the recruitment of hnRNP-K to its genomic targets [36].

Although the biological significance of lncRNAs has perhaps been most extensively investigated in cancers, it is noteworthy that several lines of evidence purport a role for lncRNAs in nonneoplastic conditions such as development [46] and cardiovascular diseases (CVDs). The first evidence suggestive of a lncRNA-CVD association stemmed from genome-wide association studies that independently identified a susceptibility locus of coronary artery disease (CAD) on human chromosome 9p21 [47, 48]. This locus is adjacent to the last exon of ANRIL. That the protein-coding genes cyclin-dependent kinase inhibitors 2A and 2B (CDKN2A and CDKN2B, resp.) lie >100 kb from associated single nucleotide polymorphisms (SNPs) suggested to the investigators that SNPs in ANRIL increases the susceptibility to CAD and other vascular diseases [49–51]. The lncRNAs MALAT1, MEG3, and TUG1 are highly expressed in endothelial cells [29] and are induced under low oxygen conditions* in vitro* in endothelial cells [29]; MALAT1 expression is similarly affected* in vivo* in ischemic limbs [29]. Inhibition of MALAT1 promoted RNA degradation in an RNase H-dependent mechanism and promoted migration of tip cells but blocked proliferation of subsequent stalk cells leading to an abnormal tube formation* in vitro* [29]. Genetic deletion or pharmacological inhibition of MALAT1 impaired vascularization* in vivo* [29]. Bioinformatics analysis of MALAT1-regulated genes revealed that MALAT1 supports the proliferation of endothelial cells through its cell cycle regulatory effects [29, 52]. Notably, the enhanced levels of MALAT1 observed in patients with ischemia [29] are consistent with the upregulation of MALAT1 previously described in* in vitro* and* in vivo* models [29].

Deep sequencing studies have identified lncRNAs in human coronary aortic smooth muscle cells (SMCs) by comparing their expression profiles to those of HUVECs [53]. After screening 31 lncRNAs, 1 lncRNA, namely, smooth muscle and endothelial cell-enriched migration/differentiation-associated long noncoding RNA (SENCR), was studied in detail, which is highly expressed in endothelial cells, SMCs, and aortic tissue [53]. In SMCs, loss of SENCR significantly enhanced SMC migration and reduced expressions of SMC contractile markers [53]. Another study evaluating the regulation and function of lncRNAs in human aortic valve cells demonstrated that cyclic stretch reduced the expression of the lncRNA HOTAIR and also that loss of HOTAIR elevated expressions of calcification-related genes, indicating its role in aortic valve calcification [54]. In the heart, Fendrr (Fetal-lethal noncoding developmental regulatory RNA) is an excellent example for the role of lncRNAs in cardiac development as intraventricular septal heart defects were observed embryonically in Fendrr-deficient mice [55].

Role of other lncRNAs in CVDs is demonstrated by lncRNA MIAT, which is associated with increased risk of myocardial infarction [56]; lncRNA ANRIL is associated with increased risk to coronary heart disease [57]; lncRNA DBE-T localizes to the facioscapulohumeral muscular dystrophy (FSHD) locus [58]; and a novel lncRNA is identified in association with HELLP syndrome (hemolysis, elevated liver enzymes, and low platelets) [59]. Furthermore, vascular lincRNA-p21 represses proliferation and induces apoptosis* in vitro* and* in vivo* in vascular smooth muscle cells [60]. Loss of endogenous lincRNA-p21 exacerbates neointima formation in injured carotid arteries in the carotid artery injury model [60]. This finding is highly relevant because it implicates lncRNAs to CVDs and indicates that lincRNA-p21 may be a novel therapeutic approach to treat human atherosclerosis and related CVDs [60].

TGF*β* belongs to a large superfamily of related polypeptides and is involved in diverse biological processes, such as cell proliferation, migration, differentiation, survival, and cell-cell and cell-matrix interaction [1]. TGF*β* plays a crucial role in the development of the cardiovascular system, affecting functions of both endothelial and periendothelial cells [61]. TGF*β*-associated signaling is a key player in metazoan biology, and its dysregulation can result in either developmental defects or other pathologies like tumor development [15]. Consequently, the output of a TGF*β*-response is known to be highly context-dependent in development, across different tissues, as well as in cancer syndromes [15]. Dysregulated TGF*β*-associated signaling is linked to human hereditary hemorrhagic telangiectasia (HHT) type II [62] and HHT type I [63]. HHT patients present with dilated blood vessels with thin walls and exhibit abnormal arteriovenous fusion and shunting. Studies have revealed that the dysregulation of the TGF*β* signaling pathway results in severe vascular abnormalities in mice models of vasculogenesis [7–12]. The TGF*β*-pathway is also responsible for the endothelial to mesenchymal transition (EndMT), a process by which endothelial cells acquire mesenchymal gene signatures to become more motile and invasive [18, 64]. EndMT plays an important role in the developmental process, as well as in the development of organ fibrosis [18, 64]. TGF*β* signaling is thus essential for vascular development and maturation, but the mechanisms of transcriptional regulation of this signaling have not been clearly defined.

To determine targets of TGF*β* in endothelial cells, we performed lncRNA and mRNA microarray analysis on total RNA isolated from TGF*β*-stimulated HUVECs. This approach allowed us to identify novel target genes of TGF*β* and provided insights into the regulation of different lncRNAs and mRNAs by TGF*β* in endothelial cells. Of the 30,584 lncRNAs screened, 2051 were significantly upregulated and 2393 were appreciably downregulated (*P* < 0.05) in response to TGF*β*1. In the same HUVEC samples, 2148 of the 26,106 mRNAs screened were upregulated and 1290 were downregulated. Interestingly, of the 2051 differentially expressed upregulated lncRNAs, MALAT1, which is highly expressed in endothelial cells [29] and is an important mediator of TGF*β* signaling [28], was the most (~220-fold) upregulated lncRNA after TGF*β*-stimulation in endothelial cells (Figure 3). The protein-coding genes LTBP3, KCNK7 and TGD3, which are adjacent to MALAT1 on chromosome 15 [27], were also significantly upregulated in our mRNA array data (Figure 3). Our data shows that 9 of the 20 lncRNAs demonstrated a direct correlation in fold-change with its associated mRNA, whereas the remaining 11 displayed an inverse correlation, which was mainly observed for the downregulated (9 out of 10) lncRNAs (Figure 3).

Pathway analysis revealed that lncRNAs upregulated in response to TGF*β*1 treatment are involved in hippo signaling, Wnt signaling, focal adhesion, neuroactive ligand-receptor interaction, and pathways specific to cancer (Table 2). The most downregulated lncRNAs are notably involved in olfactory transduction, PI3-Akt signaling, Ras signaling, neuroactive ligand-receptor interaction, and apoptosis (Table 3). The proposed common pathophysiological basis between cancer and CVDs [65–68] is strengthened by the role of lncRNAs such as MALAT1 [29, 44], p21 [49, 60], ANRIL [38, 49, 60], and HOTAIR [39, 54] in the development of cancer as well as in CVDs. Accordingly, differentially expressed lncRNA MALAT1 and pathway analysis of our data also demonstrate the common pathways indicating similar pathophysiological basis between cancer and CVDs (Table 2). Results of bioinformatics GO analysis, as described in Table 4, grouped the differentially expressed mRNAs under the following three categories: biological processes, cellular component, and molecular function. GO terms most broadly associated with upregulated mRNAs were biological function, protein binding, and signalling (Table 4). GO terms associated with downregulated mRNA were mainly enriched in cell, response to stimulus, and multicellular organism process (Table 4). This is the first lncRNA and mRNA transcriptome profile of TGF*β*-mediated changes in human endothelial cells. These observations may reveal some new targets of TGF*β* in endothelial cells and CVD-associated endothelial dysfunction. Further investigations of novel genes identified by this study will provide new clues concerning the mechanisms of vascular development by TGF*β* and contribute to therapeutic approaches to vascular diseases as well as treating cancer.

Interest in the contribution of LncRNAs to human health and disease is booming, but much effort is required to determine the full contribution and the mechanisms by which lncRNAs exert their effects. Efforts such as the Encyclopedia of DNA Elements (ENCODE) project aiming to identify all functional elements in the human genome are making major progress [69]; methods based on second-generation RNA sequencing are expected to provide a more detailed picture of the whole human lncRNA transcriptome. The lack of a complete understanding of functional motifs, low expression levels of some lncRNAs, and the need for a better definition of lncRNAs regulatory regions make the characterization of lncRNA challenging. One of the most important challenges is to identify all encoded functional lncRNAs, and emerging genomic, epigenomic, and bioinformatics approaches will be crucial in this context. However, the restricted spatiotemporal expression of many lncRNAs, as well as the binding of transcription factors to noncoding loci, could be used as evidence of functionality. The poor conservation and the fact that most lncRNAs are expressed as various transcript variants challenges the identification of specific biological functions and mechanisms of action. Often, identification of lncRNA sequences from published studies is not trivial and chromosomal localization is not provided. To avoid confusion and to facilitate the use and reproduction of the data, more details should be provided (e.g., chromosomal localization and deposition of the identified transcript into publicly available databases), which we have implemented in our data presentation. Furthermore, the mechanism of action has only been identified for a few lncRNAs.

Despite these challenges, in a short period, lncRNAs have become a major new class of transcripts that potentially comprise a major component of the genome's information content in comparison to the abundance and complexity to the proteome. LncRNAs have already been reported in a wide range of human diseases suggesting their crucial activity in human health and disease [33]. In addition, therapeutic strategies that target endogenous mRNA molecules could also be adapted to target lncRNAs, whose expression is dysregulated in human CVDs. These observations suggest that lncRNAs represent a novel and versatile class of molecules that are centrally important to the modulation of different CVD conditions and could potentially be utilized for developing novel diagnostic and therapeutic approaches to cure CVDs. With respect to the predictive value of the measured lncRNAs in human diseases, the increased MALAT1 expression levels in ischemic patients and the initial levels of ANRIL and KCNQ1OT1 in peripheral blood mononuclear cells in patients with left ventricular dysfunction at 4-month follow-up [70] suggest that lncRNAs might also be useful as indicators for CVDs. These important developments are expected in this area and exciting times lie ahead of us.

## Supplementary Material

Quality Assessment of RNA Samples and Data Analysis. RNA integrity and genomic DNA contamination were evaluated by denaturing agarose gel electrophoresis. In all of the samples, the intensity of the upper 28S ribosomal RNA band was about twice that of the lower 18S band thereby confirming the integrity of the RNA studied. The absence of smears above the 28S band attests to the purity of the RNA samples. RNA quantity and purity were also assessed with the NanoDrop ND-1000. The combination of optical density (OD) A260/A280 ratios that were close to 2.0 and A260/A230 ratios that exceeded 1.8 further confirmed the purity of the RNA used. The Box Plot is a well accepted means to quickly visualize the distribution of a dataset. Our Box plots (10th, 90th percentile) showed comparable distributions of expression values after normalization. Hierarchical clustering is a widely used method for analysis of gene expression. Cluster analysis groups samples based on their expression levels with dendrograms summarize the arrangement of said clusters.
The supplementary tables report the fold change, *P* value, false discovery rate, annotations, and raw and normalized intensities for differentially expressed and up-/down-regulated lncRNAs and mRNAs in TGF*β*1-treated HUVECs relative to control HUVECs.

## Figures and Tables

**Table 1:** 10 Most differentially expressed (up- and downregulated) lncRNAs in HUVECs upon TGF*β*1 (10 ng/mL) stimulation.

	Sequence name	RNA length	Chr.	Fold	*P* value
Upregulated lncRNAs	MALAT1	8708	15	223.69	3.72601*E* − 06
	RP11-327I22.8	1761	6	137.52	8.04408*E* − 06
	PSMD6-AS2	2555	11	110.50	1.03209*E* − 06
	BC016035	1170	18	105.83	9.96754*E* − 05
	CRNDE	659	3	105.66	2.85351*E* − 07
	RP5-1103G7.4	750	2	84.49	1.8145*E* − 05
	DA315543	538	16	64.10	2.62297*E* − 05
	TM4SF19-TCTEX1D2	1022	9	59.10	7.56202*E* − 06
	DMD-AS3	293	1	54.68	3.75391*E* − 05
	CTD-2026G22.1	1035	7	53.39	2.06995*E* − 06

Downregulated lncRNAs	AC144521.1	919	3	7.90	0.000111558
	BX114362	693	5	7.47	0.000885235
	D16471	2448	X	7.43	0.005627670
	uc.117	251	3	7.17	0.004263809
	LOC729678	2874	5	7.01	0.001343734
	LINC00593	1330	15	6.81	9.68611*E* − 05
	RP11-594C13.2	369	14	6.71	0.002673799
	LINC00494	508	20	6.64	0.008476439
	RP11-574O16.1	585	2	6.57	0.001869003
	LOC643401	490	5	6.54	9.53526*E* − 05

**Table 2:** Results of bioinformatics analyses on upregulated pathways in HUVECs after TGF*β*1 (10 ng/mL) stimulation.

Pathway analysis	Upregulated gene count	*P* value
Hippo signaling pathway	153	0.0002709
Wnt signaling pathway	139	0.0006834
Basal cell carcinoma	55	0.0017771
Hedgehog signaling pathway	51	0.0027492
Pathways in cancer	327	0.0052783
Osteoclast differentiation	131	0.0067513
Melanogenesis	101	0.0073718
Axon guidance	127	0.0095634
Pertussis	75	0.0114669
Neuroactive ligand-receptor interaction	321	0.0157052
Synaptic vesicle cycle	63	0.0158835
NOD-like receptor signaling pathway	57	0.0186762
Acute myeloid leukemia	57	0.0186762
Neurotrophin signaling pathway	120	0.0205091
Focal adhesion	206	0.0236221
Proteoglycans in cancer	225	0.0259569
Adrenergic signaling in cardiomyocytes	149	0.0279908
Notch signaling pathway	48	0.0376197
Prolactin signaling pathway	72	0.0414023
Jak-STAT signaling pathway	156	0.0438904
Cytokine-cytokine receptor interaction	271	0.0439402
Prostate cancer	89	0.0448626

**Table 3:** Results of bioinformatics analyses on downregulated pathways in HUVECs after TGF*β*1 (10 ng/mL) stimulation.

Pathway analysis	Downregulated gene count	*P* value
Olfactory transduction	405	0.0036241
Apoptosis	86	0.0050919
PI3K-Akt signaling pathway	346	0.006535
mRNA surveillance pathway	91	0.0082309
Ribosome biogenesis in eukaryotes	85	0.0119435
Circadian rhythm	30	0.0140969
Chemical carcinogenesis	80	0.0189201
Ras signaling pathway	227	0.0201366
Melanoma	71	0.0211199
Hypertrophic cardiomyopathy (HCM)	85	0.0284512
Rap1 signaling pathway	213	0.0320182
Estrogen signaling pathway	100	0.0381713
Tight junction	134	0.0382214
Nicotinate and nicotinamide metabolism	28	0.0382726
Drug metabolism-cytochrome P450	68	0.0398014
Serotonergic synapse	114	0.0453301
Neuroactive ligand-receptor interaction	321	0.0459533
Tyrosine metabolism	39	0.0463610

**Table 4:** Results of bioinformatics GO (gene ontology) enrichment analyses to determine the roles of differentially expressed mRNAs in GO terms.

	Upregulated				Downregulated			
	GO term	Count	% of total DE genes	*P* value	GO term	Count	% of total DE genes	*P* value
Biological process	Cell communication	686	41.7	5.77*E* − 06	Response to stimulus	587	55.3	1*E* − 06
	Biological regulation	1136	69.1	8.48*E* − 06	Cation transport	97	9.1	8*E* − 06
	Organ development	356	21.7	9.07*E* − 06	Multicellular organismal process	494	46.6	1*E* − 05
	Anatomical structure development	540	32.8	1.27*E* − 05	Single-multicellular organism process	478	45.1	2*E* − 05
	Signaling	674	41.0	1.6*E* − 05	Cell surface receptor signaling pathway	265	25.0	5*E* − 05

Cellular component	Plasma membrane part	273	15.5	5.43*E* − 05	Plasma membrane	380	33.9	6*E* − 09
	Neuron part	123	7.0	0.000426	Cell periphery	386	34.5	8*E* − 09
	Intrinsic component of plasma membrane	170	9.7	0.00069	Cell part	1006	89.8	0.002
	Cell projection	180	10.2	0.00236	Cell	1006	89.8	0.002
	Cell periphery	525	29.9	0.00265	Integral component of membrane	389	34.7	0.0026

Molecular function	Channel activity	68	4.2	5.66*E* − 05	Signaling receptor activity	124	12.0	3*E* − 05
	Passive transmembrane transporter activity	68	4.2	5.66*E* − 05	Receptor activity	138	13.3	9*E* − 05
	Transmembrane transporter activity	124	7.7	0.000639	Signal transducer activity	144	13.9	0.0001
	Protein binding	917	57.1	0.000751	Molecular transducer activity	144	13.9	0.0001
	Cation transmembrane transporter activity	85	5.3	0.000822	Transmembrane signaling receptor activity	113	10.9	0.0001
