# Supplementary material for: Investigation of TGFβ1-Induced Long Noncoding RNAs in Endothelial Cells
Source: Int J Vasc Med. 2016 Apr 10;2016:2459687. doi: 10.1155/2016/2459687 (PMC4842052; doi:10.1155/2016/2459687)
Supplement: Supplementary file 1 — Quality Assessment of RNA Samples and Data Analysis. RNA integrity and genomic DNA contamination were evaluated by denaturing agarose gel electrophoresis. In all of the samples, the intensity of the upper 28S ribosomal RNA band was about twice that of the lower 18S band thereby confirming the integrity of the RNA studied. The absence of smears above the 28S band attests to the purity of the RNA samples. RNA quantity and purity were also assessed with the NanoDrop ND-1000. The combination of optical density (OD) A260/A280 ratios that were close to 2.0 and A260/A230 ratios that exceeded 1.8 further confirmed the purity of the RNA used. The Box Plot is a well accepted means to quickly visualize the distribution of a dataset. Our Box plots (10th, 90th percentile) showed comparable distributions of expression values after normalization. Hierarchical clustering is a widely used method for analysis of gene expression. Cluster analysis groups samples based on their expression levels with dendrograms summarize the arrangement of said clusters. The supplementary tables report the fold change, P value, false discovery rate, annotations, and raw and normalized intensities for differentially expressed and up-/down-regulated lncRNAs and mRNAs in TGFβ1-treated HUVECs relative to control HUVECs. [file 2459687.f1.zip › 2459687.pdf]

Investigation of TGFβ1-induced Long Non-Coding RNAs in Endothelial Cells

Krishna K. Singh,[1,2,3,4] Pratiek N. Matkar,[4,5] Adrian Quan,[2] Laura-Eve Mantella,[2,6]
Hwee Teoh,[2,7] Mohammed Al-Omran[1,3,4,8], Subodh Verma[2,3,4,6]

[1]*Division of Vascular Surgery, Keenan Research Centre for Biomedical Science at St. Michael's Hospital, Toronto, Ontario, M5B 1W8, Canada*

[2]*Division of Cardiac Surgery, Keenan Research Centre for Biomedical Science at St. Michael's Hospital, Toronto, Ontario, M5B 1W8, Canada*

[3]*Department of Surgery, University of Toronto, Toronto, Ontario, M5T 1P5, Canada*

[4]*Institute of Medical Science, University of Toronto, Toronto, Ontario, M5S 1A8, Canada*

[5]*Division of Cardiology, Keenan Research Centre for Biomedical Science at St. Michael's Hospital, Toronto, Ontario, M5B 1W8, Canada*

[6]*Department of Pharmacology and Toxicology, University of Toronto, Toronto, Ontario, M5S 1A8, Canada*

[7]*Division of Endocrinology & Metabolism, Li Ka Shing Knowledge Institute at St. Michael's Hospital, Toronto, Ontario, M5B 1W8, Canada*

[8]*Department of Surgery, King Saud University and the King Saud University-Li Ka Shing Collaborative Research Program, Riyadh 12372, Kingdom of Saudi Arabia*

Correspondence should be addressed to Subodh Verma; Division of Cardiac Surgery, St. Michael's Hospital, 8th Floor, Bond Wing, 30 Bond Street, Toronto, ON, M5B 1W8, Canada, Tel.: 1-416-864-5997; Fax: 1-416-8645881; e-mail: vermasu@smh.ca

Keywords: lncRNA, endothelial cells, TGFβ1

Supplementary Figure 1

A

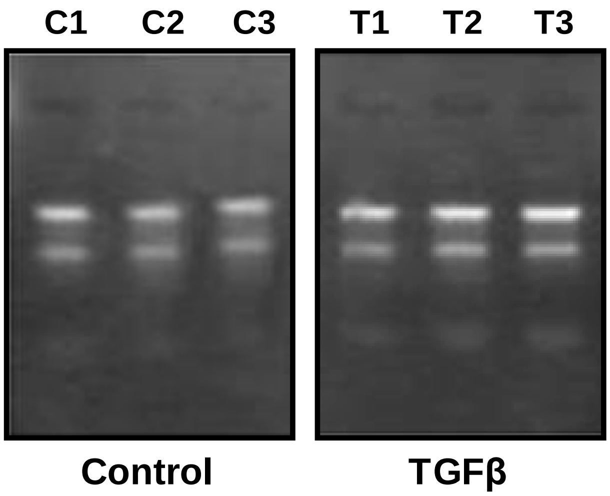

Sample	OD260/280 Ratio	OD60/230 Ratio	Concentration (ng/µl)	Volume (µl)	Quantity (ng)
C1	2.03	1.99	177.66	30	5329.8
C2	2.02	2.03	152.35	30	4570.5
C3	2.06	2.05	185.49	30	5564.7
T1	1.98	2.00	242.89	30	7286.7
T2	2.04	2.09	209.66	30	6289.8
T3	2.03	2.07	223.61	30	6708.3

B

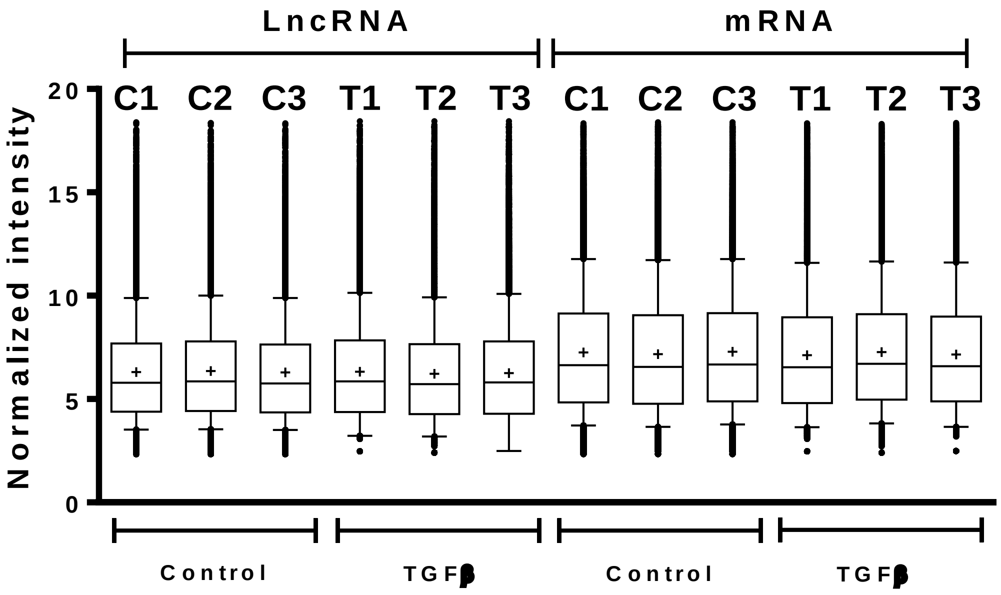

Supplementary Figure 2

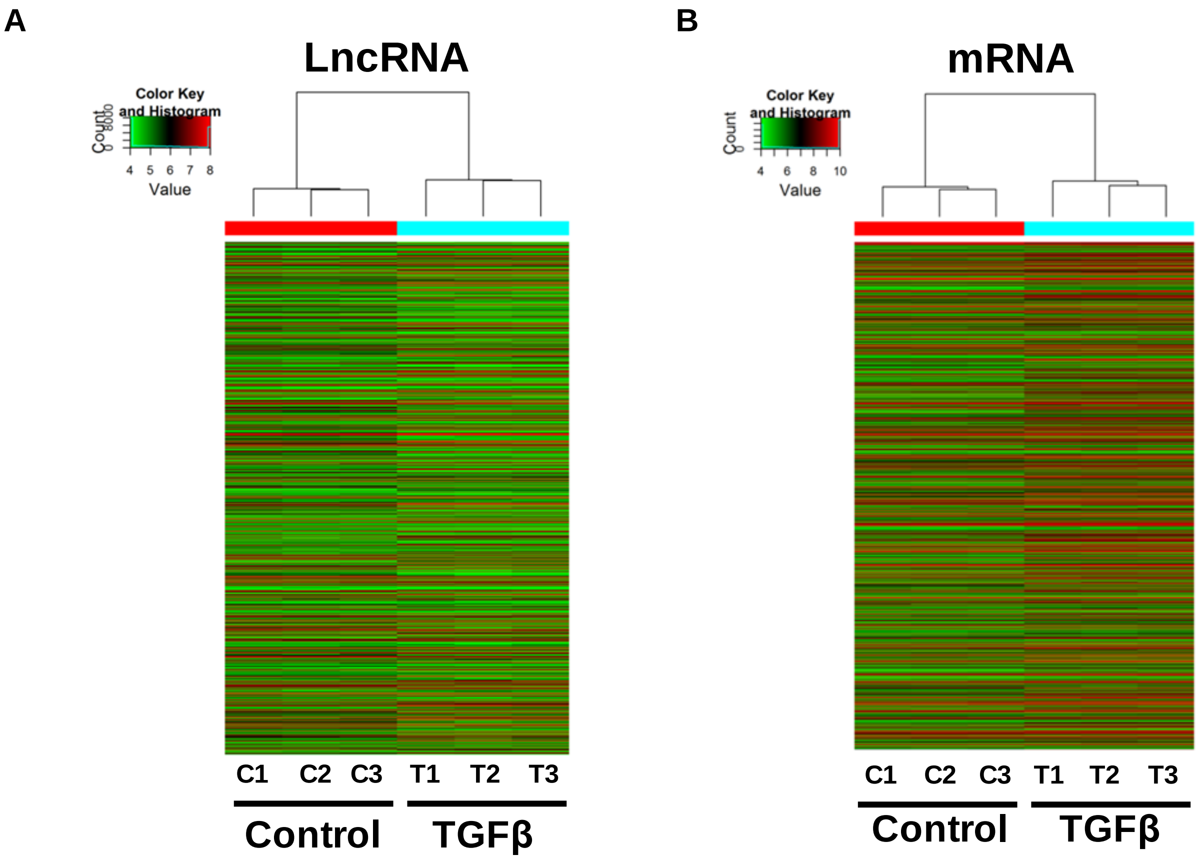

Supplementary Figure 1. Quality assessment of RNAs, lncRNAs and mRNAs Data. A. Image of denaturing agarose gel (1%) used to assess RNA integrity and genomic DNA contamination. The 28s and 18s rRNA bands were clear and intact. The larger rRNA (28s) bands were more intense in comparison to the corresponding lower rRNA (18s) bands. The table shows the absorbance ratio for wavelengths 260nm/280nm and 260nm/230nm, concentration and the quantity of RNA used for array. **B.** Box-and-Whisker plots (10th, 90th percentile) showing normalized intensity for the 6 study samples to quickly visualize the distribution of our dataset. Mean intensity is denoted with a "+" sign. C1, C2 and C3 represent the control group, and T1, T2 and T3 represent the TGFβ1 (10 ng/mL)-treated group.

Supplementary Figure 2. Heat map and hierarchical clustering of differences in lncRNA and mRNA expression from HUVECs exposed to TGFβ1 (10 ng/mL) vs. Control. A & B. The dendrogram shows the relationships among the expression levels of samples. Hierarchical clustering that was performed based on 'differentially expressed lncRNAs and mRNAs', shows a distinguishable lncRNA and mRNA expression profiling among samples.
